# Recent advances and evolving strategies in the treatment of lumbar disc herniation

**DOI:** 10.3389/fneur.2025.1706784

**Published:** 2025-12-19

**Authors:** Yun Tong, Lanying Yu, Kaifeng Luo, Xiong Yan, Ming Chen, Libin Wang

**Affiliations:** 1 Department of Pain, The Affiliated Hospital of Jiujiang University, Jiujiang, China; 2 Hospital Infection Management Department, The Affiliated Hospital of Jiujiang University, Jiujiang, China; 3 Department of Traumatology and Orthopaedics, Orthopaedics Hospital, The First Affiliated Hospital of Nanchang University, Nanchang, China

**Keywords:** artificial intelligence, future directions, lumbar disc herniation, minimally invasive surgery, regenerative medicine, treatment trends

## Abstract

Lumbar disc herniation (LDH) remains a leading cause of low back pain and sciatica, imposing a considerable global health and socioeconomic burden. Over the past decades, substantial progress has been made in both conservative and surgical interventions, yet controversies persist regarding optimal treatment strategies, long-term efficacy, and recurrence prevention. This review provides a comprehensive overview of current therapeutic approaches, including pharmacological management, physical therapy, minimally invasive and open surgical techniques, as well as emerging biological therapies. Special attention is given to platelet-rich plasma (PRP), bone marrow aspirate concentrate (BMAC), and chemonucleolysis, which demonstrate potential in delaying disc degeneration and enhancing tissue repair. Moreover, we highlight the growing role of artificial intelligence (AI) in diagnosis, surgical planning, prognosis prediction, and rehabilitation monitoring, along with the increasing emphasis on multidisciplinary team (MDT) management. Finally, we discuss ongoing challenges, such as the need for standardized long-term outcome evaluation, individualized treatment pathways, and the clinical translation of regenerative medicine. By integrating traditional strategies with novel technologies, this review underscores the evolving paradigm of LDH management toward more minimally invasive, personalized, and multidisciplinary approaches.

## 1 Introduction

Lumbar disc herniation (LDH) is one of the most common degenerative spinal disorders in clinical practice and represents a leading cause of low back pain and sciatica in adults (1). The annual incidence of LDH with nerve root involvement ranges from 0.2 to 2.7 per 1,000 adults, most frequently affecting individuals between 30 and 50 years of age. Major risk factors include high body mass index (BMI), smoking, and occupations requiring heavy lumbar loading (2). Additionally, in the context of an aging population, the role of non-communicable diseases such as type 2 diabetes and cardiovascular disease in predisposing to or exacerbating LDH is gaining recognition, potentially through mechanisms involving chronic systemic inflammation and impaired microvascular health (3). In China, more than 200 million people are affected by lumbar spine diseases, of whom approximately 15.2% suffer from LDH (4). With population aging, lifestyle changes, and increasing social stress, the incidence of LDH has shown a continuous upward trend, making it a pressing global health concern.

**Figure 1:**
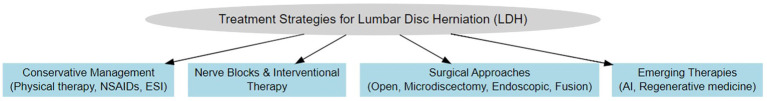
Treatment strategies for lumbar disc herniation.

In clinical practice, the therapeutic strategies for LDH have evolved toward diversification, encompassing conservative treatment, never block, interventional procedures, surgical approaches and emerging therapies (Figure 1) (5). Current guidelines recommend conservative management as the first-line therapy, including non-steroidal anti-inflammatory drugs (NSAIDs), physical rehabilitation, and functional exercise. Most patients achieve significant symptom relief within 6 to 8 weeks. For those who fail conservative treatment or present with neurological deficits, timely minimally invasive or open surgery is indicated, followed by early rehabilitation to facilitate functional recovery and improve quality of life (6, 7). Although traditional open surgery can effectively decompress neural structures, it is increasingly limited by its invasiveness, long recovery period, and higher risk of complications (8). In recent years, advances in medical technology have fostered the development of minimally invasive techniques and artificial intelligence–assisted interventions, offering new prospects for precision treatment and functional restoration in LDH (9, 10). Meanwhile, both international and domestic guidelines are being continuously updated, placing greater emphasis on multidisciplinary collaboration and individualized patient management.

Despite significant progress in LDH management, challenges remain in patient stratification, recurrence prevention, long-term outcome evaluation, and clinical translation of novel technologies. Therefore, a systematic review of recent advances and evolving strategies, together with an exploration of future directions, is of great significance for optimizing clinical decision-making and improving patient prognosis. This review aims to summarize the major developments, key strategies, and future trends in LDH treatment, providing theoretical insights and practical guidance for clinical practice and further research.

## 2 Advances in conservative treatment

### 2.1 Pharmacological therapy

Pharmacological therapy remains a cornerstone of conservative management for lumbar disc herniation (LDH), aimed at alleviating pain and facilitating functional recovery. Contemporary best practice advocates for a stratified management strategy, where treatment is aligned with the predominant pain phenotype—distinguishing between nociceptive/inflammatory and neuropathic pain—and its intensity. This approach is supported by international guideline recommendations (11, 12).

For nociceptive inflammatory pain, non-steroidal anti-inflammatory drugs (NSAIDs) are the most commonly prescribed first-line agents, demonstrating efficacy in alleviating low back and leg pain with a good short-term safety profile (13). However, evidence from systematic reviews indicates their overall effect is limited, a view reflected in some high-quality guideline recommendations advising against their routine use for acute radiculopathy.

A significant proportion of LDH-related radicular pain encompasses a neuropathic component. While anticonvulsants like gabapentin are no longer routinely recommended, agents specifically targeting neuropathic pain—such as pregabalin, duloxetine (an SNRI), or tricyclic antidepressants (e.g., amitriptyline)—are recommended for patients with predominant neuropathic features. This recommendation is informed by international guideline syntheses, which assign fair to optional recommendation grades to these agents (12), and finds a pathophysiological correlate in evidence suggesting shared mechanisms between chronic pain and affective disorders, as observed in preclinical models (14). Therapy must be individualized and carefully titrated.

For severe acute pain inadequately controlled by other measures, opioids may be prescribed for a strictly limited duration due to well-documented risks of dependence and adverse effects (15). Importantly, the decision to initiate opioid therapy should consider the broader treatment pathway. A large-scale retrospective cohort study (*n* = 573,082) demonstrated that among patients with LDH, those managed non-surgically had the highest one-year cumulative opioid burden and the highest percentage of opioid users at follow-up (9.6%). In contrast, patients who underwent early surgical intervention (within 30 days of diagnosis) had consistently lower long-term average daily morphine milligram equivalents (MME), a lower incidence of opioid use (6.1%), and a lower cumulative MME burden compared to both non-surgical patients and those who underwent late surgery (after 30 days). This suggests that delaying definitive surgical management, when indicated, may inadvertently contribute to higher long-term opioid exposure (16). This cautious approach is reinforced by recent high-quality clinical guidelines, which recommend against their routine use.

Recent research supports several alternative and adjunctive pharmacological strategies within this stratified framework. Systemic corticosteroid therapy, exemplified by a single intravenous dose of methylprednisolone (500 mg), has shown significant and sustained pain relief superior to conventional NSAIDs in one study, with benefits persisting at six-month follow-up (17). This finding is corroborated by a direct comparative clinical trial in post-discectomy patients, where intramuscular betamethasone provided superior early analgesia compared to pregabalin or ibuprofen (18). Furthermore, epidural steroid injections are recognized as effective short-term interventions for radicular pain, as supported by guideline reviews. Topical agents, such as Baimai ointment evaluated in a multicenter RCT, offer a favorable safety profile and have been shown to reduce pain scores by approximately 40% within 2 weeks (19).

**Figure 2:**
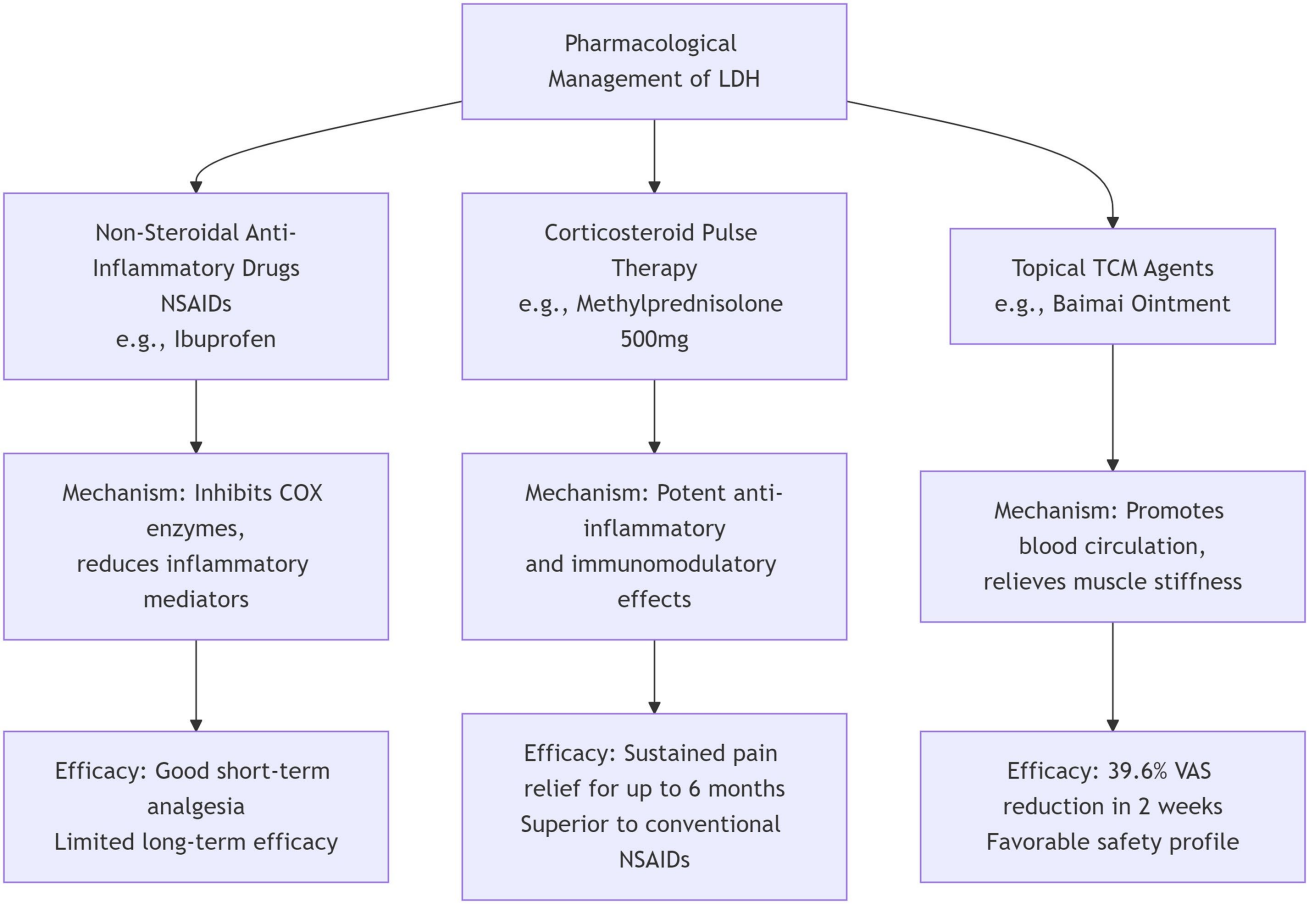
Pharmacological management of lumbar disc herniation.

In conclusion, a stratified, phenotype-guided approach is essential for optimizing the pharmacological management of LDH (Figure 2). The therapeutic spectrum ranges from first-line NSAIDs and targeted neuropathic agents to short-term opioids, systemic steroids, and topical therapies. Future research should focus on refining this personalized framework and developing novel therapeutic agents.

### 2.2 Physical therapy and exercise rehabilitation

Physical therapy represents a cornerstone of conservative management for LDH, encompassing diverse modalities such as core stabilization training, traction, manual therapy, mobilization, and Pilates. Among these, core stabilization exercises strengthen the paraspinal and abdominal musculature, enhance spinal stability, and thereby effectively relieve pain and improve functional status. A meta-analysis has shown that mechanical traction reduces intradiscal pressure, enlarges the intervertebral foramen, and alleviates nerve root compression. Compared with conventional physiotherapy, it is more effective in reducing pain and Oswestry Disability Index (ODI) scores, though less effective in improving spinal mobility, suggesting that it should be combined with other interventions (20).

Pilates, which integrates core stabilization with flexibility training, has demonstrated beneficial effects in pain relief, trunk control, and quality of life improvement (21). Regarding manual therapy, studies indicate that spinal mobilization (MOB) combined with neural mobilization (NM) is superior to spinal manipulation therapy (SMT) combined with NM in improving symptoms and function, with more pronounced long-term outcomes (22).

Conventional physiotherapy approaches—including electrical stimulation, deep massage, and stability training—also yield notable improvements in pain relief and reduction of herniated disc volume. Non-surgical spinal decompression therapy (NSDT) may promote disc resorption, but its additional benefits for symptom relief and functional improvement are limited, emphasizing the importance of multimodal rehabilitation strategies (23).

Systematic exercise interventions (≥2 weeks in duration, ≥2 sessions per week, moderate to low-intensity bodyweight training) significantly reduce lumbar and leg pain, improve ODI and Japanese Orthopaedic Association (JOA) scores, and enhance spinal mobility. Mechanistically, such interventions may restore disc and neural function by strengthening spinal stability, improving microcirculation, and modulating immune mediators such as TNF-α, IL-6, IL-1β/IL-17, and IL-10 (24). Furthermore, core muscle stabilization and strengthening exercises have been shown to provide robust benefits in alleviating pain and improving functional impairment in LDH patients, with strong clinical recommendations (25).

Recent studies have also indicated that repetitive spinal magnetic stimulation (SMS), as a non-invasive neuromodulation technique, shows potential in treating neuropathic pain associated with lumbar degenerative diseases. For instance, a randomized, double-blind, placebo-controlled trial demonstrated that a single session of SMS significantly reduced pain in patients with lumbar spondylosis, with effects sustained up to the fourth day post-treatment (26). High-intensity laser therapy (HILT) has been shown to effectively relieve pain and improve function in patients with lumbar disc herniation (27). HILT significantly reduces early postoperative pain and improves functional outcomes by modulating inflammatory responses, inhibiting NF-κB pathway and proinflammatory cytokines such as TNF-*α* and IL-8, while enhancing tissue repair through mitochondrial cytochrome c oxidase activation and ATP restoration (28).

In summary, physical therapy and exercise rehabilitation play a central role in relieving pain, enhancing function, optimizing vertebral stability, and delaying degenerative progression. Future studies should further explore the synergistic mechanisms of multimodal interventions and refine individualized rehabilitation pathways.

### 2.3 Traditional Chinese Medicine (TCM) therapy

As an essential component of conservative management for LDH, Traditional Chinese Medicine (TCM) has achieved increasing evidence-based recognition in recent years. Modern studies suggest that herbal medicine may promote spontaneous resorption of herniated discs and alleviate nerve root irritation by modulating inflammatory mediators, enhancing angiogenesis, and improving microcirculation.

High-quality randomized controlled trials (RCTs) using MRI to quantify herniated disc volume have verified that herbal medicine combined with exercise intervention provides significant advantages in reducing disc size and relieving pain (29). Certain herbal formulations have also demonstrated superior analgesic efficacy compared to conventional pharmacological agents, with a lower incidence of adverse effects.

Acupuncture, when used in conjunction with traditional rehabilitation, has also shown notable benefits. A multicenter retrospective study reported that acupuncture significantly increased the cross-sectional area of paraspinal muscles such as the erector spinae and multifidus, reduced fatty infiltration, and yielded greater improvements in VAS and JOA scores compared with conventional rehabilitation (30). These findings indicate that acupuncture not only provides analgesic effects but may also enhance spinal stability by improving muscle quality, making it an important adjunct to conservative LDH management.

Additionally, the concept of “inflammation-preserving therapy,” which is reflected in TCM interventions, emphasizes harmonizing qi and blood and promoting the body’s self-repair rather than suppressing the inflammatory response. Prospective studies have demonstrated that this approach may facilitate natural disc resorption and reduce recurrence risk (31).

TCM interventions not only relieve symptoms but also focus on regulating the overall condition and restoring the local microenvironment, offering a treatment paradigm with distinct Chinese characteristics for LDH. Future research should emphasize high-quality, multicenter RCTs to further clarify the mechanisms and target populations of TCM therapies.

### 2.4 Future challenges and directions in conservative management

The future of conservative treatment for lumbar disc herniation will increasingly focus on the dual modulation of inflammatory responses. Current studies indicate that spontaneous resorption of the nucleus pulposus relies on macrophage-mediated inflammatory processes, with an overall resorption rate reaching 76.6%, while pro-inflammatory cytokines (e.g., TNF-α, IL-6) are positively correlated with pain levels, and anti-inflammatory cytokines (e.g., IL-4, IL-10) are associated with pain relief (32, 33). This suggests that future conservative treatment must move beyond simple anti-inflammatory suppression toward precisely regulating macrophage polarization and cytokine balance to achieve both pain control and promotion of resorption. Additionally, developing individualized treatment prediction models by integrating imaging features and biomarkers, along with advancing high-quality clinical research, will be key to optimizing conservative treatment strategies.

## 3 Advances in interventional and minimally invasive treatments

### 3.1 Epidural steroid injection (ESI)

Epidural steroid injection (ESI) is a commonly used conservative intervention for alleviating radicular pain associated with LDH, particularly in the acute phase of radiating leg pain (34). The major injection routes include transforaminal (TFESI), caudal (CESI), and interlaminar (ILES) approaches (35).

TFESI is considered superior in pain relief and functional improvement because it allows precise drug delivery to the affected nerve root. Compared with other approaches, TFESI requires fluoroscopic or ultrasound guidance to ensure accurate targeting and to minimize complications such as vascular or neural injury (36). Studies have shown that TFESI significantly improves pain scores (e.g., NRS, ODI, FRI) as well as cognitive performance (e.g., PALT, COWAT, PASAT). Moreover, reductions in serum miRNA-155 levels following TFESI suggest that its analgesic and neuroregulatory effects may be mediated through epigenetic mechanisms (37).

In terms of durability, short-term follow-ups confirm that steroid injections provide superior analgesia compared with local anesthetics or saline during the first month post-treatment. However, at 3–12 months, no significant differences were observed among treatment groups regarding functional improvement, indicating that ESI is more suited for short-term symptom control rather than long-term therapeutic substitution (38). Clinical outcomes of TFESI also vary with disc herniation type. One study reported that patients with paramedian LDH achieved greater pain relief and larger improvements in VAS scores compared to those with foraminal LDH, with a lower incidence of complications, suggesting higher safety and suitability in this subgroup (39).

When comparing ESI routes, TFESI generally outperforms CESI and ILES, particularly in analgesia and functional recovery. Within ILES, the parasagittal approach provides better drug distribution and sustained effects than the traditional midline approach, indicating its potential advantages in selected cases (40, 41).

To further optimize clinical application, predictive models have been developed to identify key factors influencing TFESI outcomes, including pain duration, imaging characteristics, and psychological status. Cost-effectiveness analyses also support TFESI as a valuable conservative intervention that provides a preoperative buffer before surgery (42). Overall, ESI—particularly TFESI—can significantly relieve radicular symptoms in the short term, offering patients a temporary reprieve before surgical intervention. However, its long-term efficacy remains limited, and clinical decisions should be tailored according to patient conditions and therapeutic goals.

### 3.2 Platelet-rich plasma (PRP) injection therapy

Platelet-rich plasma (PRP), as an emerging biological therapy, has gained increasing attention in the non-surgical management of LDH in recent years. Derived from autologous blood, PRP is prepared by concentrating platelet components enriched with growth factors, which possess anti-inflammatory, neuroregenerative, and tissue-repairing properties. Multiple studies have demonstrated that PRP injections are superior to conventional epidural steroid injections in alleviating radicular leg pain and improving functional outcomes such as the Oswestry Disability Index (ODI) and Visual Analogue Scale (VAS) scores, with a lower incidence of adverse events, thereby highlighting its favorable safety and tolerability profile (43, 44). As an autologous product, PRP also avoids the potential complications of steroids, including immunosuppression, osteoporosis, and neurotoxicity, making it particularly suitable for patients who respond poorly to traditional therapies or have contraindications.

Prospective clinical studies further confirmed that transforaminal PRP injection for LDH significantly improved multiple evaluation indices, including VAS, ODI, and the Straight Leg Raise Test (SLRT), with therapeutic effects lasting up to 12 months and no serious complications reported (45). Moreover, PRP has emerged as a promising alternative to steroid injections, offering comparable or even more durable analgesic and functional benefits, thereby underscoring its value in the management of chronic discogenic low back pain (35).

When combined with minimally invasive surgery, PRP has shown additional therapeutic potential. For instance, transforaminal endoscopic lumbar discectomy (TELD) combined with PRP significantly improved postoperative VAS and ODI scores at 3–12 months, reduced recurrence rates, and enhanced spinal canal cross-sectional area and disc morphology, suggesting that PRP contributes to disc reconstruction and stabilization (46). Similarly, percutaneous endoscopic lumbar discectomy (PELD) with PRP demonstrated sustained improvements in radiological parameters (e.g., disc height, canal area) and clinical outcomes, further supporting its potential to delay disc degeneration and promote tissue regeneration (47).

In summary, PRP represents a safe and effective biological therapy with regenerative potential, offering a novel option for non-surgical treatment of LDH. Its advantages in clinical efficacy, patient satisfaction, and recurrence control highlight the need for further clinical promotion and in-depth research.

### 3.3 Nerve block

Nerve block, as a key component of multimodal analgesia strategies, has been increasingly applied in the non-surgical management of LDH (48). By selectively interrupting specific neural pathways, nerve block can effectively relieve radicular pain, reduce postoperative opioid consumption, enhance patient comfort, accelerate functional recovery, and lower the risk of chronic pain development.

A retrospective study enrolled 64 patients with L4–5 disc herniation who received either surgical intervention or nerve block, and assessed visual analogue scale (VAS) scores and muscle strength (MRC) at baseline, 1, 6, and 12 months post-treatment, alongside MRI measurements of disc protrusion length, area, and spinal canal occupancy. Results indicated that surgical intervention provided superior pain relief at all follow-up time points; however, the difference in low back pain between the two groups was no longer statistically significant at 12 months. Further ROC analysis identified disc protrusion lengths of 6.31 mm and 6.23 mm as predictive thresholds for successful outcomes with surgery and nerve block, respectively (49).

Technological advances have markedly improved the precision and safety of nerve block procedures. Studies have shown that ultrasound-guided transforaminal selective nerve root block (SNRB) is superior to traditional blind techniques, offering greater accuracy and safety. It also reduced procedural time and significantly improved VAS, Japanese Orthopaedic Association (JOA) scores, Pain Rating Index (PRI), and Present Pain Intensity (PPI) scores at 30 min, 1 week, and 3 months post-treatment, without any serious complications, thereby demonstrating a favorable safety profile (50).

Moreover, a prospective study further validated the clinical utility of SNRB in patients with LDH-related radicular pain. The intervention provided sustained reductions in numeric rating scale (NRS) and Oswestry Disability Index (ODI) scores within 3 months, while also improving psychological well-being and overall quality of life (51). These findings indicate that nerve block serves not only as an effective option for patients unresponsive to conservative therapy but also as a safe and valuable analgesic strategy for those unsuitable for surgical intervention.

In summary, nerve block demonstrates promising short-term efficacy in LDH-related pain management, particularly with the aid of advanced imaging guidance. Its enhanced precision and safety support its broader clinical adoption.

### 3.4 Future directions in interventional and minimally invasive therapies

Regenerative medicine therapies, such as mesenchymal stem cell (MSC) and PRP injections, represent a promising frontier in the minimally invasive management of chronic low back pain. A systematic review and meta-analysis evaluated the long-term efficacy of these biological treatments across various spinal targets, including intervertebral discs, epidural space, facet joints, and sacroiliac joints (52). The study found that intradiscal injections of both MSCs and PRP showed Level III evidence for pain relief and functional improvement, while epidural, facet joint, and sacroiliac joint injections presented Level IV evidence. Despite these encouraging results, the authors emphasized the current scarcity of high-quality randomized controlled trials and the predominance of observational studies with significant heterogeneity. Future research should prioritize well-designed RCTs with standardized protocols, larger sample sizes, and longer follow-up periods to establish the safety, optimal dosing, and long-term effectiveness of these regenerative approaches. Additionally, further investigation is needed to clarify the mechanisms of action, identify the most responsive patient subgroups, and compare regenerative therapies directly with conventional interventions such as steroid injections or surgery.

## 4 Surgical strategies

### 4.1 Indications and timing for surgery

Surgical intervention for LDH should be based on well-defined indications to maximize therapeutic efficacy while avoiding unnecessary risks. Clinically, the major surgical indications for LDH include: (1) significant or progressive neurological deficits, such as muscle weakness or sensory loss, indicating severe nerve root compression; (2) concomitant cauda equina syndrome characterized by acute symptoms including bladder and bowel dysfunction or perineal numbness, necessitating urgent surgical intervention; and (3) failure of standardized conservative treatment for at least 6 weeks, with persistent severe pain or functional impairment significantly affecting quality of life. In addition to symptomatology, factors such as patient age, overall health status, degree of disc degeneration, and imaging findings should be comprehensively considered to formulate individualized surgical decisions.

Evidence suggests that for patients with predominant sciatica, surgical intervention is recommended only when symptoms persist for more than 6–12 weeks without improvement, or when new neurological deficits develop (53). In particular, the presence of bladder or bowel dysfunction warrants decompression surgery within 24–48 h to maximize the likelihood of recovery of urinary and defecatory functions. The timing of surgery also plays a critical role in neurological recovery. For patients with moderate-to-severe motor weakness (MRC ≤ 3/5), surgical intervention within 3 days of symptom onset increases the complete recovery rate from approximately 50 to 97%. Even in cases of mild weakness (MRC 4/5), surgery within 8 days raises the recovery rate from 75 to 98%.

These findings highlight the crucial importance of early recognition and timely surgical intervention in optimizing outcomes. Surgical indications for LDH should therefore be determined by a comprehensive assessment of symptom severity, neurological status, and response to conservative therapy, while precise timing of surgery directly influences neurological recovery and quality of life—underscoring the need for accurate evaluation and individualized treatment strategies.

### 4.2 Main surgical techniques

Traditional open discectomy (OD) is primarily indicated for patients with complex anatomical variations or concomitant spinal stenosis. Although effective, it is limited by greater surgical trauma and prolonged recovery. In contrast, microdiscectomy (MED), currently the most widely adopted minimally invasive technique, allows precise removal of herniated nucleus pulposus under microscopic visualization. It offers advantages such as smaller incision, reduced blood loss, and faster recovery, while achieving clinical outcomes comparable to OD (54, 55).

Building upon these advancements, percutaneous transforaminal endoscopic discectomy (PTED) further minimizes surgical trauma and hospital stay, though it requires a higher level of technical expertise. Endoscopic minimally invasive surgery, with its high-definition magnified view and continuous irrigation, effectively reduces intraoperative bleeding and complication rates, shortens hospitalization, and alleviates postoperative pain, thereby demonstrating favorable safety and efficacy. However, no significant differences have been observed between endoscopic and non-endoscopic procedures regarding functional recovery and operative time, and the high equipment cost continues to limit its widespread adoption (56).

Long-term follow-up studies have shown that percutaneous endoscopic lumbar discectomy (PELD) provides greater pain relief and functional improvement than open fenestration discectomy (OFD) in both early and final follow-up, while also delaying disc degeneration and preserving segmental stability, with no significant differences in recurrence rates between the two techniques (57). A meta-analysis comparing discectomy and sequestrectomy reported no significant differences in recurrence, reoperation, or complication rates; however, sequestrectomy demonstrated superior relief of sciatica and low back pain at one- and two-year follow-ups, suggesting potential advantages in long-term efficacy (58).

### 4.3 Comparison of surgical efficacy and safety

**Figure 3:**
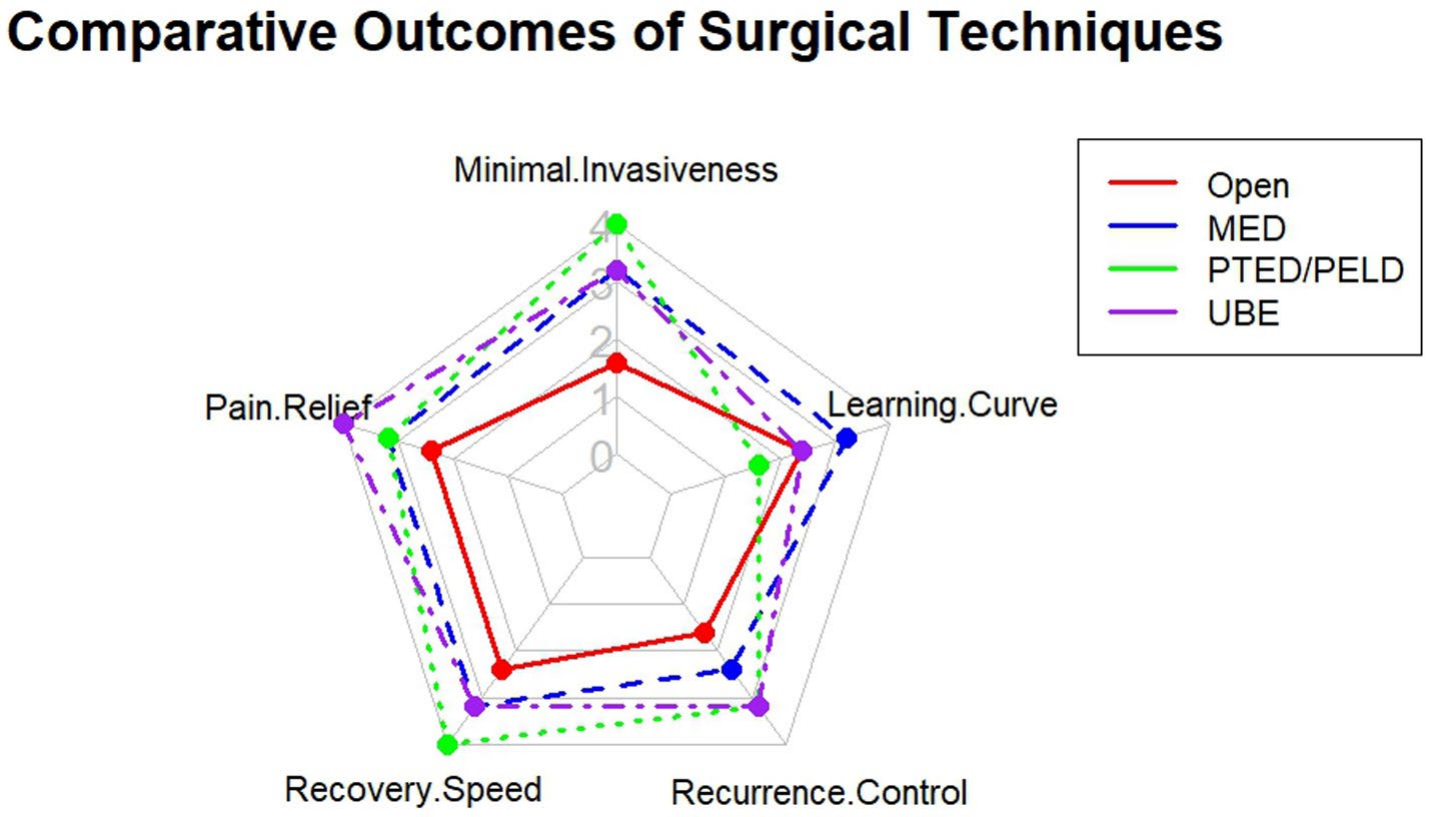
Comparative visualization of key surgical outcomes across different techniques for treating lumbar disc herniation.

A comprehensive comparison of the efficacy, safety, and technical characteristics of various surgical procedures is crucial for clinical decision-making. As visually compared in Figure 3, techniques such as MED, PTED/PELD, and UBE offer distinct trade-offs in terms of invasiveness, learning curve, pain relief, and recurrence control.

Multiple evidence-based studies have demonstrated that patients with LDH who undergo early surgical intervention within 6 months of symptom onset achieve significant improvements in radicular pain and quality of life, while also reducing the risk of chronicity and irreversible functional deficits. In contrast, patients with chronic symptoms or prolonged waiting times exhibit less postoperative improvement. The choice of surgical technique should therefore be individualized, taking into account the patient’s clinical condition, radiological classification, and personal needs.

A meta-analysis including 23 studies with 4,068 patients compared the efficacy and safety of various minimally invasive procedures such as PTED, MED, PEID, MD, FED, PELD, and PELD combined with autologous PRP injection. The results indicated that all procedures effectively relieved pain and improved function. PTED showed advantages over MED in terms of operative time and hospital stay but was associated with a slightly higher recurrence rate. PEID provided marginally superior functional outcomes and pain relief compared with PTED while being technically less demanding. Notably, PELD combined with PRP demonstrated the best outcomes in terms of back pain relief and recurrence reduction (59). In addition, both PETD and unilateral biportal endoscopy (UBED) were shown to be safe and effective for symptom relief, with UBED offering a shorter learning curve and comparable improvements in postoperative pain and complication rates compared to PETD (60).

Enzymatic chemonucleolysis (including chymopapain, condoliase, and collagenase) has emerged as a minimally invasive non-surgical option, with an overall treatment success rate of approximately 79%, significantly superior to placebo and comparable to surgical outcomes. The incidence of serious adverse events was relatively low, with condoliase and collagenase demonstrating better safety profiles than chymopapain. Furthermore, the proportion of patients requiring subsequent surgery showed no significant difference, supporting the role of chemonucleolysis as a promising non-surgical alternative (61).

In recent years, unilateral biportal endoscopy (UBE) has gained increasing attention due to its minimally invasive advantages. Studies have shown that proficiency in UBE requires approximately 43 cases to overcome the learning curve. Careful case selection is recommended during the initial phase, and conventional microsurgical techniques should be retained as backup options until sufficient surgical expertise is achieved (62).

### 4.4 Postoperative recurrence and complication management

Although advances in both minimally invasive and open surgical techniques have markedly reduced the risk of complications, issues such as postoperative recurrence, disc degeneration, and nerve root adhesion remain clinically relevant. Management strategies for recurrent lumbar disc herniation (rLDH) include repeat minimally invasive or open discectomy, complemented by postoperative rehabilitation and secondary prevention measures. Key to preventing complications are standardized surgical procedures, precise patient selection, and comprehensive perioperative management.

Studies have identified independent risk factors for recurrence following percutaneous endoscopic lumbar discectomy (PELD), including diabetes mellitus, protruded-type herniation, advanced age, Modic changes, smoking, obesity, heavy manual labor, and limited surgeon experience. In contrast, factors such as surgical approach, disc segment, and herniation location showed no significant association with recurrence (63). Additional studies have highlighted intraoperative determinants, including the integrity of nucleus pulposus removal, annular condition, and segment-specific risks at L4–L5, which together with systemic factors form a multidimensional predictive framework for recurrence. This provides a basis for individualized risk assessment and targeted interventions (64). Zhou et al. (65) further confirmed Modic changes as a high-risk factor and proposed disc fragmentation as a novel intraoperative indicator to guide more thorough debridement of pathological tissue.

In terms of postoperative pain management, a prospective study demonstrated that LDH patients who received appropriate preoperative opioid therapy achieved superior early and long-term pain outcomes compared with opioid-naïve patients. This suggests that opioids may serve as a short-term adjunctive analgesic option in severe cases, although potential side effects and addiction risks must be carefully monitored (66).

Regarding procedure-specific complications, sacral epiduroscopic lumbar decompression (SELD) was associated with an overall complication rate of approximately 6.3%, mainly due to incomplete decompression (2.4%) and recurrent herniation (1.6%). Importantly, complication rates decreased significantly with surgical experience, falling from 12% in the first 50 cases to 2.6% in the subsequent 77 cases. Risk was notably higher at the L4–L5 segment compared with L5–S1 (67). For lumbar disc herniation treated with minimally invasive transforaminal lumbar interbody fusion (MIS-TLIF) versus open TLIF, the overall complication rates were comparable. However, wound healing problems were more frequently observed in the open TLIF group, while no severe complications such as dural tears or implant failure occurred in either cohort (68).

In elderly patients undergoing single-level surgery, those aged ≥80 years exhibited significantly higher risks of postoperative complications compared with middle-aged patients, particularly in terms of pulmonary embolism, transfusion requirements, urinary tract infection, and 30-day mortality. Nonetheless, the overall complication risk remained within a manageable range, with higher ASA classifications further amplifying risk (69).

### 4.5 Evolution and future trends in surgical strategies

The future of surgery for lumbar disc herniation is characterized by intelligent precision and procedural integration. The next significant advancement will be the strategic convergence of surgical platforms, such as merging the robust three-dimensional visualization of tubular systems with the ultra-minimal access of endoscopic techniques, to create more versatile and less disruptive solutions (70).

Simultaneously, surgical decision-making is evolving from experience-based to algorithm-driven. The emergence of evidence-based selection models, which utilize specific radiographic features to objectively guide the choice of surgical approach, exemplifies this shift towards standardized and personalized planning to optimize outcomes (71).

## 5 Advances and future trends

### 5.1 Emerging technologies and biological therapies

Electroacupuncture combined with Shenxie Analgesic Capsules has recently been explored as a novel conservative treatment strategy for chronic sciatica caused by LDH. High-quality randomized controlled trials are underway to systematically evaluate its long-term analgesic efficacy and functional benefits. Preliminary clinical findings suggest that this combined therapy not only effectively alleviates chronic sciatica and improves functional status and quality of life, but also carries fewer adverse effects. It may represent a safe, cost-effective, and efficient alternative to nonsteroidal anti-inflammatory drugs (NSAIDs), thereby expanding the therapeutic options for patients with chronic sciatica (72).

Chemonucleolysis, a minimally invasive treatment for LDH, has undergone substantial progress in recent years. Initially performed with single-agent collagenase, the approach has evolved to include combination regimens with collagenase, ozone, and anti-inflammatory or analgesic drugs to enhance outcomes. In terms of imaging guidance, traditional C-arm fluoroscopy is gradually being replaced by multidetector CT, which offers more precise localization and improved safety. Furthermore, diverse injection routes (e.g., transforaminal, intradiscal, and extraforaminal) provide greater flexibility for patients with varying LDH types and severity. Clinical studies report long-term efficacy rates of up to 90%, with a 6-month success rate as high as 95% and a relatively low incidence of complications. However, challenges remain regarding drug dosage standardization and patient selection criteria. Improper intradiscal injection may result in disc height loss or nerve root compression. Future research should prioritize standardized protocols, optimized patient screening, and advanced imaging guidance to broaden the clinical application of this technique (73).

In addition, bone marrow aspirate concentrate (BMAC), owing to its immunomodulatory properties and multipotent differentiation potential, has been shown to improve disc degeneration and relieve pain, although clinical challenges remain regarding cell survival and optimal timing of therapy. Low-intensity pulsed ultrasound (LIPUS), by promoting soft tissue regeneration and enhancing cell proliferation, has demonstrated potential for disc repair in animal models, but its efficacy in clinical settings requires further validation (74).

### 5.2 Digital health technologies

Digital health technologies have shown increasing promise across the entire diagnostic and therapeutic spectrum of LDH, with notable applications in imaging-based diagnosis, surgical planning, risk prediction, and rehabilitation strategies. By leveraging big data and machine learning models, these technologies have the potential to enhance diagnostic accuracy, promote individualized treatment, and improve surgical precision and safety.

In rehabilitation, AI-assisted prediction has been particularly valuable. Studies have demonstrated that machine learning models such as logistic regression and gradient boosting machines (GBM) can effectively predict patient outcomes following physical therapy. The GBM model achieved an AUC of 0.936, with prediction accuracy exceeding 80% once patients had completed more than five therapy sessions. Such predictive models facilitate early identification of poor responders to conservative treatment, enabling optimization of individualized rehabilitation strategies and improving both efficiency and patient satisfaction (75).

Supervised deep learning algorithms have also been applied to forecast functional prognosis. By integrating baseline characteristics such as age, symptom duration, SF-36 scores, and VAS pain scores, these models can predict patients’ Oswestry Disability Index (ODI) at 6 months with a mean absolute error as low as 5.9%, and as low as 1.5% in the best-performing models. This capability not only identifies candidates likely to benefit from conservative treatment, reducing unnecessary surgeries, but also flags individuals requiring early surgical intervention, thus supporting the principle of “early recognition and early intervention” (76).

Complementing these predictive approaches, digital health technologies also enable dynamic functional assessment. For example, integrating wearable surface electromyography (sEMG) with a Random Forest machine learning algorithm allows for the localization of compressed nerve roots (L5 vs. S1) in LDH patients through gait analysis. This model demonstrated an accuracy of 84% (AUC 0.93), showcasing how the fusion of wearable sensors and AI can translate real-time biomechanical data into a quantitative diagnostic aid, providing functional insights that complement static imaging (77).

Similarly, in the domain of gait analysis and functional monitoring, wearable inertial measurement units (IMUs) combined with machine learning offer a powerful tool for objective assessment. A recent study utilized an adaptive Kalman filter to fuse accelerometer and gyroscope data from two shank-mounted IMUs, extracting a comprehensive set of 12 gait parameters (including spatiotemporal, kinematic, and stability metrics). A subsequent Multilayer Perceptron (MLP) classifier achieved 95.50% accuracy in distinguishing the gait patterns of LDH patients from healthy subjects. This approach provides a quantitative, low-cost method for continuously monitoring functional deterioration and rehabilitation progress outside the laboratory, representing a key component of remote patient management in digital health (78).

Meanwhile, convolutional neural network (CNN)-based AI systems have been deployed in imaging diagnostics, automatically identifying key surgical indicators on MRI (e.g., herniation size >6 mm, nerve root compression). These systems achieved a diagnostic accuracy of 94.7%, outperforming human evaluation alone by approximately 3% while reducing evaluation time by 23%. Importantly, the “human–AI collaboration” model yielded the highest diagnostic accuracy, underscoring the potential of digital tools in clinical decision support. However, risks of misclassification remain, highlighting the need for integration with clinical data and multimodal assessments (79).

Notably, large language models (LLMs) such as ChatGPT have been explored for generating diagnostic and therapeutic recommendations for radiculopathic LDH. Current versions, however, demonstrate limited alignment with established guidelines (e.g., ChatGPT-4 achieved only 59% concordance with NASS recommendations), with frequent overgeneralization (45%), omission of information (28%), and fabricated citations. Although LLMs may provide supplemental insights—such as highlighting the lower infection rates of outpatient surgical centers—they require rigorous validation and cautious use before clinical integration (80).

The applications of digital health technologies in LDH management are gradually transitioning from research to clinical practice. Future development should focus on improving model generalizability and interpretability, enhancing multimodal data integration, and strengthening physician–technology collaboration to achieve truly intelligent and individualized care.

### 5.3 Multidisciplinary management

**Figure 4:**
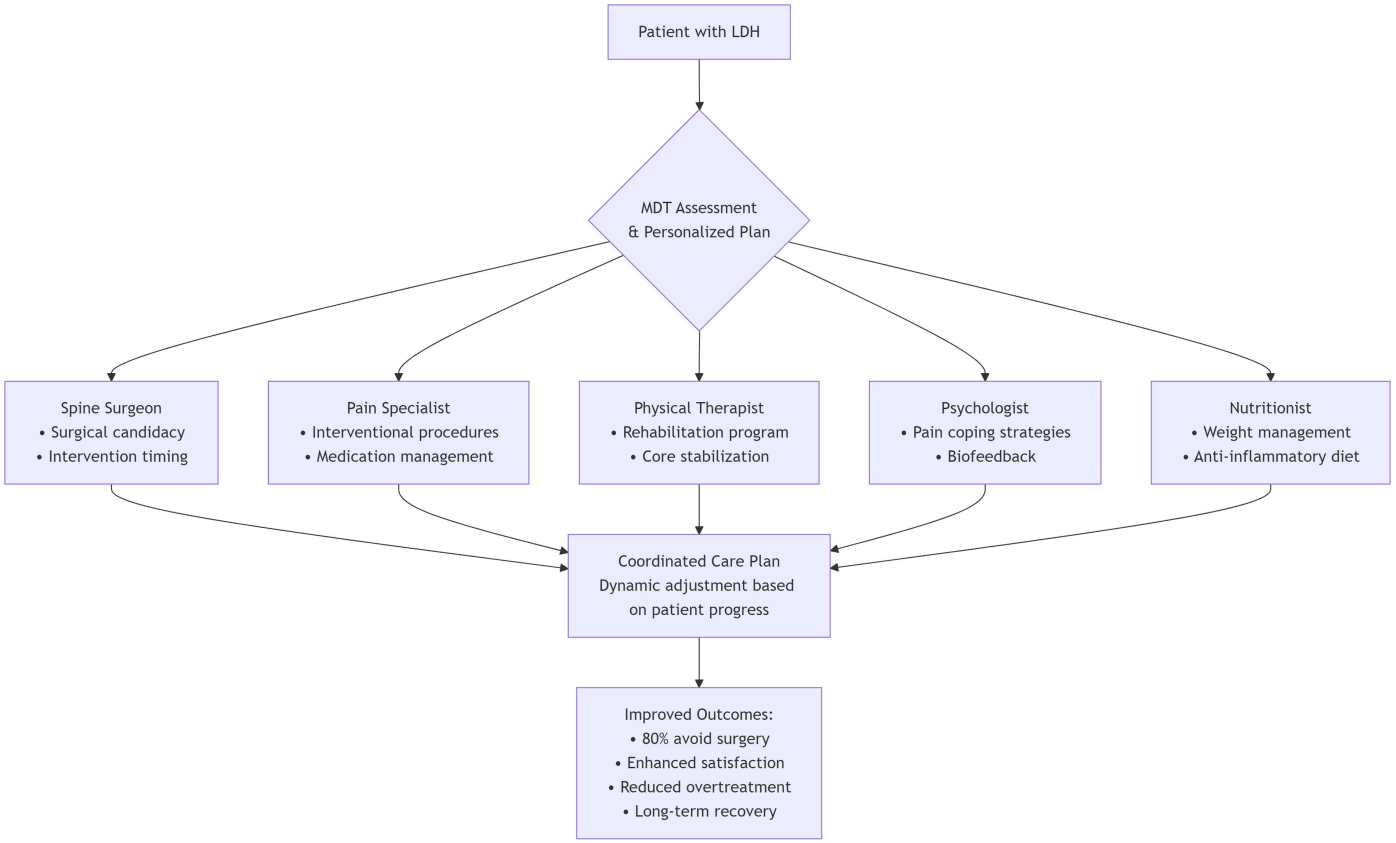
Multidisciplinary team (MDT) management pathway for lumbar disc herniation.

With evolving international guidelines, the management paradigm for LDH has shifted from single-modality interventions toward integrated, multidimensional strategies. Multidisciplinary team (MDT) management, incorporating evidence-based input from surgery, rehabilitation, pain medicine, psychiatry, and nutrition, is increasingly recognized as essential for tailoring dynamic, individualized treatment plans that meet diverse patient needs (Figure 4) (81, 82). MDT-based approaches have been shown to improve efficiency, enhance patient satisfaction, and reduce overtreatment and redundant investigations. One study reported that LDH patients receiving inpatient MDT-based conservative therapy achieved significantly greater pain relief and neurological improvement compared with those treated using conventional single-modality strategies. Notably, nearly 80% of patients achieved long-term recovery without requiring surgical intervention (83).

MDT emphasizes early identification of surgical candidates and non-responders to conservative therapy, while coordinating pain control, functional rehabilitation, and psychological support. This approach is particularly suited for chronic cases with comorbidities and complex needs. Integrating psychological interventions is crucial, as kinesiophobia and fear avoidance have been identified as significant barriers to recovery in older adults with chronic pain. A scoping review revealed that kinesiophobia is a stronger determinant of physical activity limitation than pain itself in this population, yet targeted interventions remain underdeveloped (84). Therefore, embedding structured psychological support within the MDT framework—addressing maladaptive fear beliefs and promoting graded activity—is essential for optimizing functional outcomes, especially in frail or elderly patients.

In the future, MDT is expected to play an increasingly prominent role in LDH treatment, especially in the domains of individualized therapy, cost control, and long-term health management. It may ultimately serve as a standardized management pathway for chronic low back pain and other degenerative spinal conditions (85).

## 6 Research prospects and challenges

Despite significant progress in LDH management—driven by advances in minimally invasive techniques, individualized treatment strategies, and multidisciplinary collaboration—important clinical and research challenges remain.

First, consensus is lacking regarding the long-term efficacy and safety of various treatment modalities, particularly with respect to differences in outcomes between younger and older patients, recurrence rates, and functional recovery. Large-scale, high-quality, long-term clinical studies are needed to establish robust evidence.

Second, achieving individualized and precision medicine remains a critical goal. Current evaluation systems rely heavily on imaging and clinical symptoms while underestimating psychosocial factors, pain perception variability, and emerging biomarkers. Future research should integrate artificial intelligence, machine learning, and bioinformatics to develop predictive models and data-driven individualized care pathways.

Third, regenerative approaches such as stem cell transplantation and tissue engineering have shown promise in preclinical studies, but their clinical translation faces major challenges, including sourcing, ethical concerns, immune rejection, and validation of efficacy. Standardized, widely applicable protocols have yet to be established.

Additionally, postoperative management and recurrence prevention require greater emphasis. Rehabilitation programs, lifestyle modifications, and long-term follow-up systems are essential to slow or prevent further degeneration. However, rehabilitation practices currently lack standardization and vary considerably across regions and institutions.

In summary, future LDH research should emphasize cross-disciplinary collaboration, mechanistic exploration, and clinical translation, advancing toward more minimally invasive, intelligent, and personalized treatment paradigms. The ultimate goal is to achieve efficient, precise, and sustainable clinical management.
